# Ovarian function recovery in breast cancer patients receiving adjuvant anastrozole treatment: updated results from the phase 3 DATA trial

**DOI:** 10.1007/s10549-024-07411-w

**Published:** 2024-06-28

**Authors:** Senna W. M. Lammers, Sandra M. E. Geurts, Karlijn E. P. E. Hermans, Irene E. G. van Hellemond, Astrid C. P. Swinkels, Carolien H. Smorenburg, Maurice J. C. van der Sangen, Judith R. Kroep, Aafke H. Honkoop, Franchette W. P. J. van den Berkmortel, Wilfred K. de Roos, Alexander L. T. Imholz, Ingeborg J. H. Vriens, Vivianne C. G. Tjan-Heijnen, on behalf of the Dutch Breast Cancer Research Group (BOOG) for the DATA investigators

**Affiliations:** 1 Department of Medical Oncology, Maastricht University Medical Centre, GROW, Maastricht University, P.O. Box 5800, 6202 AZ Maastricht, the Netherlands; 2 Department of Medical Oncology, Catharina Hospital, Eindhoven, the Netherlands; 3 Clinical Research Department, Netherlands Comprehensive Cancer Organisation (IKNL), Nijmegen, the Netherlands; 4 Department of Medical Oncology, Netherlands Cancer Institute, Amsterdam, the Netherlands; 5 Department of Radiation Oncology, Catharina Hospital, Eindhoven, the Netherlands; 6 Department of Medical Oncology, Leiden University Medical Centre, Leiden, the Netherlands; 7 Department of Medical Oncology, Isala Clinics, Zwolle, the Netherlands; 8 Department of Medical Oncology, Zuyderland Medical Centre Heerlen-Sittard-Geleen, location Sittard-Geleen, Geleen, the Netherlands; 9 Department of Surgery, Gelderse Vallei Hospital, Ede, the Netherlands; 10 Department of Medical Oncology, Deventer Hospital, Deventer, the Netherlands

**Keywords:** Breast neoplasms, Aromatase inhibitors, Chemotherapy-induced amenorrhea, Chemotherapy-induced ovarian function failure, Ovarian function recovery

## Abstract

**Purpose:**

Patients with chemotherapy-induced ovarian function failure (CIOFF) may experience ovarian function recovery (OFR). Earlier, we showed that OFR during treatment with anastrozole impacted the prognosis of hormone receptor-positive (HR+) breast cancer (BC) patients with CIOFF. Here, we present the long-term follow-up results.

**Methods:**

Postmenopausal women with HR+ BC who were 45–57 years of age and received chemotherapy were identified from the phase 3 DATA study (NCT00301457) on the extended use of anastrozole. Eligible patients were categorised into two groups: patients with CIOFF and definitely postmenopausal patients. Patients with CIOFF were monitored for OFR. Disease-free survival (DFS), distant recurrence-free survival (DRFS), and overall survival (OS) were compared between patients with OFR and patients without OFR using multivariable Cox regression analyses, including OFR as a time-dependent covariate. BC-specific mortality (BCSM) was compared between groups using the Fine and Gray method.

**Results:**

This study included 656 patients: 395 patients with CIOFF and 261 definitely postmenopausal patients. OFR occurred in 39 (12%) of 329 patients with CIOFF who were monitored for OFR. The median follow-up time was 13.3 years. Patients with OFR experienced a deterioration in DFS (hazard ratio (HR) = 1.54; 95% confidence interval (CI) 0.85–2.81), DRFS (HR = 1.51; 95% CI 0.73–3.11), OS (HR = 1.64; 95% CI 0.75–3.55), and BCSM (subdistribution HR = 1.98; 95% CI 0.84–4.63) when compared with patients without OFR.

**Conclusion:**

In patients with CIOFF, OFR during treatment with anastrozole was associated with a deterioration in BC outcomes. These findings underscore the importance of adequate ovarian function suppression in this subgroup of patients.

**Supplementary Information:**

The online version contains supplementary material available at 10.1007/s10549-024-07411-w.

## Introduction

Endocrine therapy is an important aspect of treatment of patients diagnosed with early-stage hormone receptor-positive (HR+) breast cancer (BC). The type of endocrine therapy is primarily based on menopausal status. In premenopausal women, endocrine therapy generally consists of a combination of ovarian function suppression (OFS) and either tamoxifen or an aromatase inhibitor for at least 5 years [1]. In postmenopausal women, aromatase inhibitors, either upfront or sequentially after 2–3 years of tamoxifen, are considered superior to tamoxifen during the first 5 years of treatment [2].

Patients who are premenopausal at diagnosis of BC may become postmenopausal as a result of chemotherapy-induced ovarian function failure (CIOFF) [3–5]. In a previous exploratory analysis of the DATA study, a randomised controlled trial evaluating the extended use of anastrozole, we showed that disease outcomes of patients with CIOFF do not differ from those of definitely postmenopausal patients of the same age [6]. Prior studies have however shown that patients with CIOFF may experience ovarian function recovery (OFR) during treatment with an aromatase inhibitor [7–11]. In the previous analysis of the DATA study, we revealed that 12% of patients with CIOFF developed OFR during treatment with anastrozole, even though chemotherapy was given two to three years earlier and patients had a median age of 48 years at randomisation [6, 11]. Importantly, at a median follow-up of 7.3 years, we showed that patients with OFR experienced a worse distant recurrence-free survival (DRFS) and overall survival (OS) when compared with patients without OFR [6].

The current study aims to update the prior results of the DATA study with six additional years of follow-up information. We first assess whether the long-term disease outcomes of patients who developed CIOFF remain similar to those of patients who were considered definitely postmenopausal. Subsequently, as the primary objective of this study, we compare the long-term disease outcomes of CIOFF patients who experienced OFR with those of CIOFF patients who did not experience OFR during treatment with anastrozole.

## Methods

### Study population

Patients were identified from the DATA study (NCT00301457): a phase 3, randomised controlled trial, which compared the efficacy of 6 versus 3 years of anastrozole in postmenopausal women with HR+ BC who were disease-free after two to three years of adjuvant treatment with tamoxifen [12, 13]. The final study population consisted of 1860 patients who were recruited from 79 hospitals in the Netherlands between 2006 and 2009.

For the current analysis, all patients aged 45–57 years at randomisation who received (neo)adjuvant chemotherapy were identified and categorised into two groups: (1) patients with CIOFF and (2) definitely postmenopausal patients [6, 11]. The following exclusion criteria were applied: the use of a gonadotropin-releasing hormone (GnRH) agonist before randomisation and the lack of postmenopausal oestradiol (E2) levels at randomisation.

### Data collection and definitions

Patients were categorised as experiencing CIOFF when the last menstrual bleeding was reported within one year before the start of chemotherapy and postmenopausal E2 levels were present at randomisation. Patients were categorised as definitely postmenopausal when the last menstrual bleeding was reported more than one year before the start of chemotherapy or a bilateral ovariectomy was performed before randomisation.

In patients with CIOFF, E2 and follicle-stimulating hormone (FSH) levels were measured every six months during the first 30 months after randomisation to monitor for the incidence of OFR, which was defined by either a return of menstrual bleeding and/or the presence of premenopausal E2 and FSH levels. Local reference values from all participating hospitals were used to define pre- and postmenopausal E2 and FSH levels. In patients with OFR, treatment adjustments were based on the decision of the treating physician.

All study participants were monitored for disease recurrence and death every 6 months in the first 6 years after randomisation and yearly thereafter. A mammogram was performed once a year. Database lock: March 7, 2022.

### Endpoints

The primary endpoint was disease-free survival (DFS), defined as time from randomisation until the occurrence of any of the following events: (non-)invasive BC recurrence, (non-)invasive second primary (breast) cancer other than basal cell or squamous cell carcinoma of the skin or carcinoma in situ of the cervix, or death from any cause. Secondary endpoints included DRFS, OS, BC-specific mortality (BCSM), and other-cause mortality (OCM). A period of DRFS ended following the development of a distant recurrence or death from any cause, whilst a period of OS ended following death from any cause. All BC-related deaths were included as events in the analysis of BCSM, whereas all non-BC-related deaths were included as events in the analysis of OCM.

### Statistical analysis

Baseline characteristics of the study population were compared using the Chi-squared test for categorical variables and the Mann–Whitney *U* test for continuous variables.

Disease outcomes of patients with CIOFF were compared with those of definitely postmenopausal patients from randomisation onwards. Survival curves for DFS, DRFS, and OS were estimated with the Kaplan–Meier method. Mortality curves for BCSM and OCM were estimated with the cumulative incidence function, thereby considering non-BC-related death (BCSM) and BC-related death (OCM) as competing events. Differences between groups were examined with the log-rank test and the Gray’s test. Patients without an event were censored at the last follow-up date in all analyses.

In addition, multivariable Cox regression analyses were performed to evaluate whether patients with CIOFF experienced any differences in, respectively, DFS, DRFS, and OS when compared with definitely postmenopausal patients. The proportional hazards assumption was tested. The Fine and Gray method was used for BCSM and OCM, thereby considering, respectively, OCM and BCSM as competing events. Confounding factors included tumour size, nodal status, histological grade, and hormone receptor status. Age at last menstruation and body mass index (BMI) were excluded as confounding factors to avoid multicollinearity with postmenopausal status. Missing values of confounding factors were imputed.

Disease outcomes of patients with OFR were compared with those of patients without OFR, using the same statistical methods as described above. Multivariable analyses included OFR as a time-dependent covariate and excluded age as a confounding factor to avoid multicollinearity with OFR. In addition, for graphical presentation, a landmark analysis starting at 1 year after randomisation was performed to compare the disease outcomes of patients with OFR during the first year of treatment with those of patients without OFR during the first year of treatment. A landmark of one year was chosen, because the majority of OFR events occurred during the first year [11]. Patients who developed an endpoint event during the first year after randomisation were excluded from the landmark analysis.

All *p* values were two-sided and considered statistically significant at a value of ≤ 0.05. All statistical analyses were performed with SPSS (version 28), Stata (version 17), and RStudio (version 2023).

## Results

**Fig. 1:**
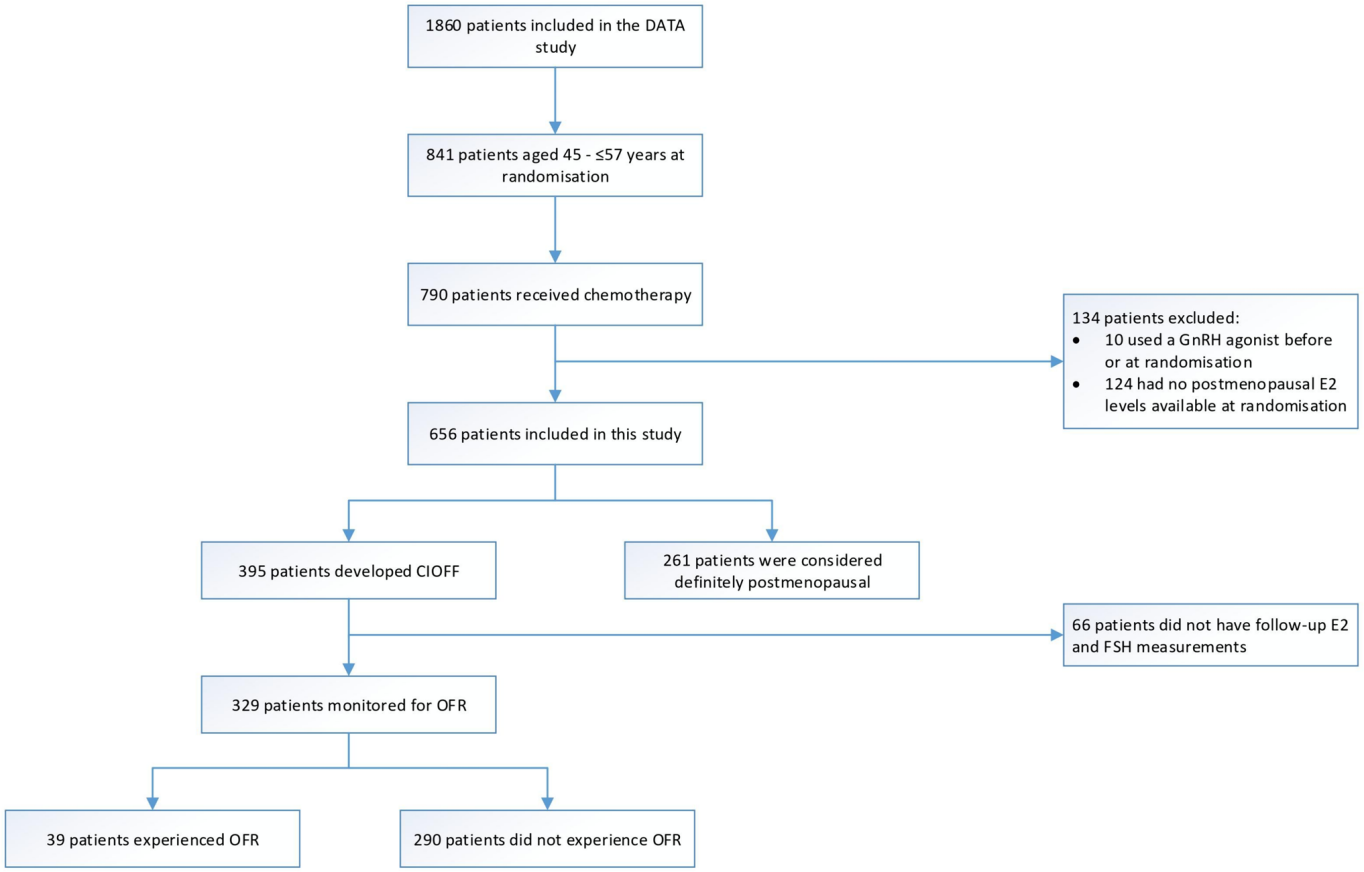
Flowchart of included patients. *CIOFF* chemotherapy-induced ovarian function failure, *E2* oestradiol, *FSH* follicle-stimulating hormone, *GnRH* gonadotropin-releasing hormone, *OFR* ovarian function recovery

In the current study, 656 patients were included: 395 patients with CIOFF and 261 patients who were considered definitely postmenopausal at randomisation (Fig. 1). Amongst the 395 patients with CIOFF, 329 patients had follow-up E2 and FSH measurements available to monitor for the incidence of OFR. These patients were considered eligible for the analysis assessing the prognostic impact of OFR. Overall, 39 patients experienced OFR, whereas 290 patients did not experience OFR during the first 30 months after randomisation. Menstrual bleeding was reported in 19 (49%) of 39 patients with OFR [6, 11]. Treatment adjustments were performed in 27 (69%) of 39 patients with OFR: 12 patients received additional treatment with a GnRH agonist, seven patients underwent a bilateral ovariectomy, six patients switched to tamoxifen, and two patients received monotherapy with a GnRH agonist [6, 11]. All patients with a BC recurrence received treatment adjustments [6].

### Baseline characteristics

The age of patients with CIOFF and patients who were considered definitely postmenopausal was similar at randomisation (median age: 51 years) (Table 1). The age at last menstruation (median age: 45 versus 48 years) was however by definition lower in patients who were already definitely postmenopausal, whereas the BMI was higher (BMI of ≥ 25 kg/m^2^: 60% versus 49%) when compared with patients with CIOFF. Definitely postmenopausal patients were more frequently diagnosed with node-negative disease (36% versus 26%), histological grade 3 tumours (38% versus 26%), and tumours expressing only one hormone receptor (26% versus 18%) when compared with patients with CIOFF.

**Table 1:** Baseline characteristics of included patients (N (%))

	All patients (*N* = 656)			Patients with CIOFF (N = 329)^a^		
	Patients with CIOFF (*N* = 395)	Definitely postmenopausal patients (*N* = 261)	*p* value	Patients with OFR (*N* = 39)	Patients without OFR (*N* = 290)	*p* value
Age at randomisation			0.96			< 0.001
Median—years (IQR)	51 (49–53)	51 (48–55)		48 (46–50)	51 (49–53)	
45–50 years	134 (34)	89 (34)		28 (72)	92 (32)	
≥ 50 years	261 (66)	172 (66)		11 (28)	198 (68)	
Age at last menstruation			< 0.001			< 0.001
Median—years (IQR)	48 (46–50)	45 (43–48)		45 (44–48)	48 (46–50)	
BMI			0.02			0.16
< 25 kg/m^2^	194 (51)	101 (40)		23 (59)	137 (49)	
25–29.9 kg/m^2^	127 (33)	104 (41)		14 (36)	95 (34)	
≥ 30 kg/m^2^	60 (16)	49 (19)		2 (5)	46 (17)	
Pathological tumour status			0.52			0.88
T1	165 (42)	106 (41)		15 (39)	122 (42)	
T2	181 (46)	129 (49)		20 (51)	136 (47)	
T3/4	49 (12)	26 (10)		4 (10)	32 (11)	
Pathological nodal status			0.005			0.35
Negative	102 (26)	94 (36)		13 (33)	76 (26)	
Positive	293 (74)	167 (64)		26 (67)	214 (74)	
Histological grade			0.004			0.13
Grade 1	72 (19)	33 (13)		4 (11)	55 (19)	
Grade 2	213 (55)	124 (49)		19 (50)	157 (55)	
Grade 3	100 (26)	96 (38)		15 (40)	72 (25)	
Hormone receptor status			0.01			0.87
ER+ and PR+	323 (82)	192 (74)		32 (82)	241 (83)	
ER+ or PR+	72 (18)	69 (26)		7 (18)	49 (17)	
HER2 status			0.38			0.29
Negative	382 (97)	257 (99)		39 (100)	279 (97)	
Positive	10 (3)	4 (2)		0 (0)	8 (3)	
Type of (neo)adjuvant chemotherapy			0.24			0.88
Anthracycline and taxane	47 (12)	27 (10)		4 (10)	38 (13)	
Anthracycline without taxane	332 (84)	230 (88)		34 (87)	239 (82)	
Taxane without anthracycline	2 (1)	1 (< 1)		0 (0)	1 (< 1)	
Other	14 (4)	3 (1)		1 (3)	12 (4)	
Recommended treatment duration of anastrozole			0.81			0.90
3 years	202 (51)	131 (50)		21 (54)	153 (53)	
6 years	193 (49)	130 (50)		18 (46)	137 (47)	

Percentages may not add up to 100% because of rounding

Missing values: age at last menstruation (*n* = 35), BMI (*n* = 21), histological grade (*n* = 18), and HER2 status (*n* = 3)

*BMI* body mass index, *CIOFF* chemotherapy-induced ovarian function failure, *E2* oestradiol, *ER* oestrogen receptor, *FSH* follicle-stimulating hormone, *HER2* human epidermal growth factor receptor-2, *IQR* interquartile range, *OFR* ovarian function recovery, *PR* progesterone receptor

^a^In 66 patients with CIOFF, no follow-up E2 and/or FSH measurements were available to monitor for the incidence of OFR

Amongst patients with CIOFF, baseline characteristics of patients with versus without OFR were comparable, except that patients with OFR were younger at randomisation (median age: 48 versus 51 years) and last menstruation (median age: 45 versus 48 years).

### Association between CIOFF and disease outcomes in the total study population

The median follow-up time was 13.3 years (interquartile range (IQR) 12.5–14.0), during which 193 DFS events, 136 DRFS events, and 116 OS events were reported (Table 2, Supplementary Table 1).

**Table 2:** Univariable and multivariable analyses of disease-free survival, distant recurrence-free survival, overall survival, breast cancer-specific mortality, and other-cause mortality

	Univariable analyses		Multivariable analyses^a^	
	(s)HR (95% CI)	*P* value	(s)HR (95% CI)	*P* value
All study participants (*n* = 656)				
Disease-free survival (*n* = 193 events)				
CIOFF vs definitely postmenopausal	0.82 (0.61–1.08)	0.16	0.79 (0.59–1.06)	0.12
Distant recurrence-free survival (*n* = 136 events)				
CIOFF vs definitely postmenopausal	0.81 (0.58–1.13)	0.22	0.79 (0.56–1.12)	0.18
Overall survival (*n* = 116 events)				
CIOFF vs definitely postmenopausal	0.75 (0.52–1.08)	0.12	0.73 (0.50–1.06)	0.10
Breast cancer-specific mortality (*n* = 73 events)				
CIOFF vs definitely postmenopausal	0.93 (0.58–1.47)	0.74	0.93 (0.56–1.54)	0.78
Other-cause mortality (*n* = 43 events)				
CIOFF vs definitely postmenopausal	0.55 (0.30–1.00)	0.05	0.48 (0.26–0.88)	0.02
Patients with CIOFF who had follow-up E2 and FSH measurements (*n* = 329)				
Disease-free survival (*n* = 81 events)				
OFR vs no OFR	1.59 (0.88–2.88)	0.13	1.54 (0.85–2.81)	0.16
Distant recurrence-free survival (*n* = 55 events)				
OFR vs no OFR	1.67 (0.82–3.41)	0.16	1.51 (0.73–3.11)	0.26
Overall survival (*n* = 44 events)				
OFR vs no OFR	1.80 (0.84–3.87)	0.13	1.64 (0.75–3.55)	0.21
Breast cancer-specific mortality (*n* = 31 events)				
OFR vs no OFR	2.36 (1.02–5.47)	0.05	1.98 (0.84–4.63)	0.12
Other-cause mortality (*n* = 13 events)				
OFR vs no OFR	0.68 (0.09–5.19)	0.71	0.78 (0.10–6.08)	0.81

In the analyses of breast cancer-specific mortality, we reported sHR instead of HR

*CI* confidence interval, *CIOFF* chemotherapy-induced ovarian function failure, *E2* oestradiol, *FSH* follicle-stimulating hormone, *(s)HR* (subdistribution) hazard ratio, *OFR* ovarian function recovery

^a^Analyses were adjusted for tumour size, nodal status, histological grade, and hormone receptor status

**Fig. 2:**
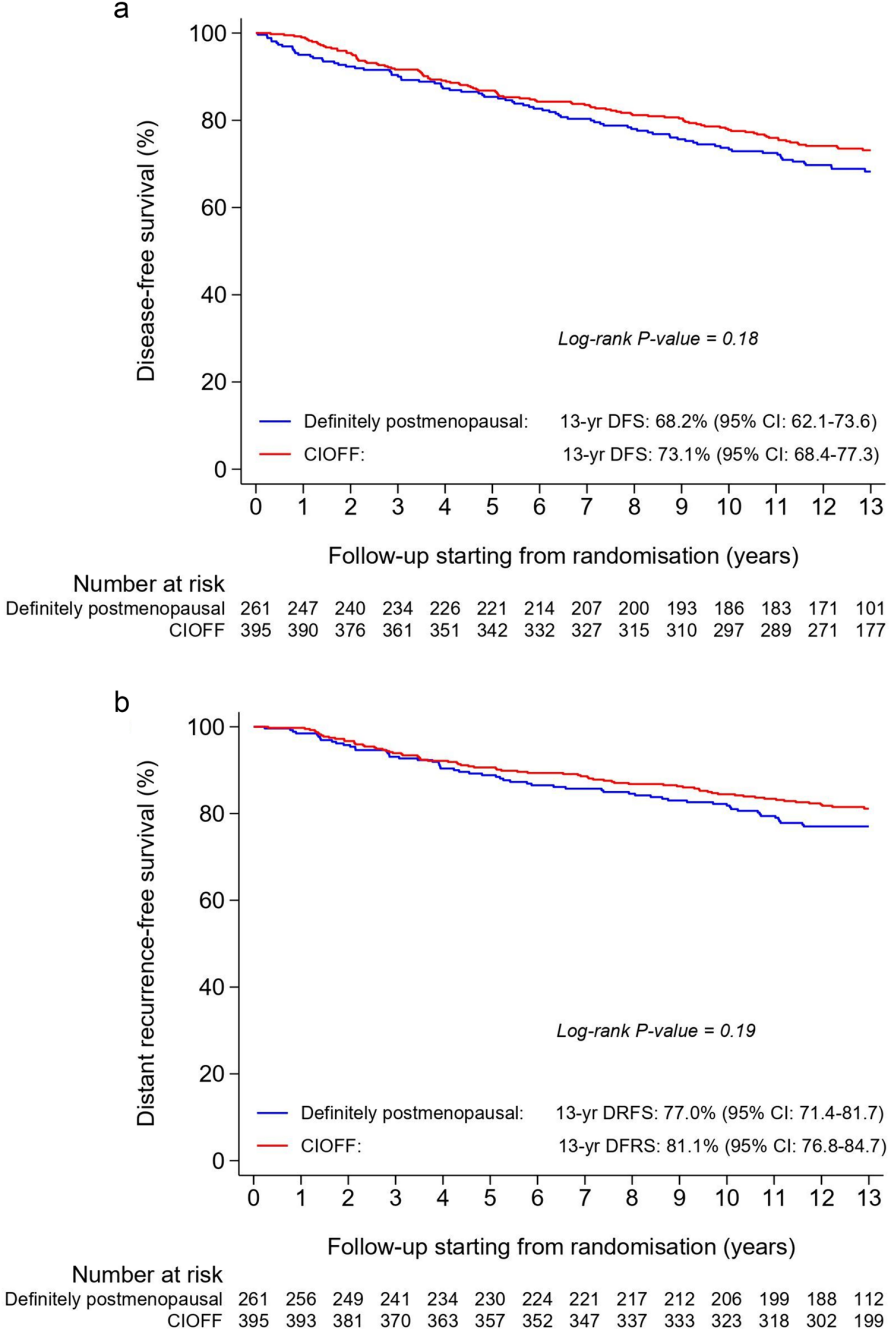

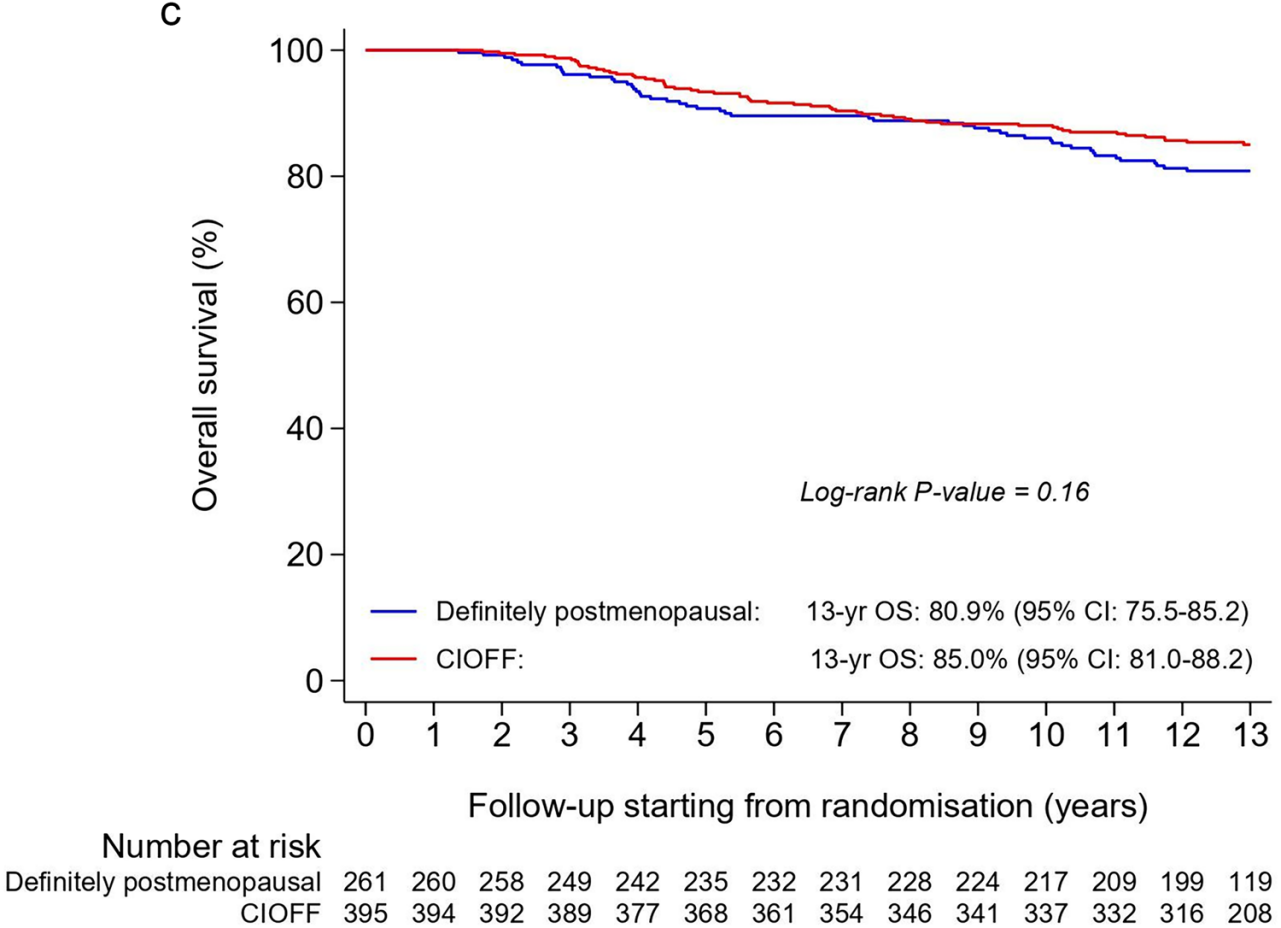
Disease-free survival (**a**), distant recurrence-free survival (**b**), and overall survival (**c**) in patients with chemotherapy-induced ovarian function failure (CIOFF) and definitely postmenopausal patients, from randomisation onwards

The 13-year DFS rate of patients with CIOFF [73.1% (95% confidence interval (CI) 68.4–77.3)] was higher than the 13-year DFS rate of definitely postmenopausal patients [68.2% (95% CI 62.1–73.6)], but this difference was not statistically significant in the multivariable analysis (hazard ratio (HR) = 0.79; 95% CI 0.59–1.06; *p* = 0.12) (Fig. 2a, Table 2).

A higher 13-year DRFS rate was also observed in patients with CIOFF [81.1% (95% CI 76.8–84.7)] versus patients who were definitely postmenopausal [77.0% (95% CI 71.4–81.7)], but again results were not statistically significant after adjustment for potential confounding factors (HR = 0.79; 95% CI 0.56–1.12; *p* = 0.18) (Fig. 2b, Table 2).

**Fig. 3:**
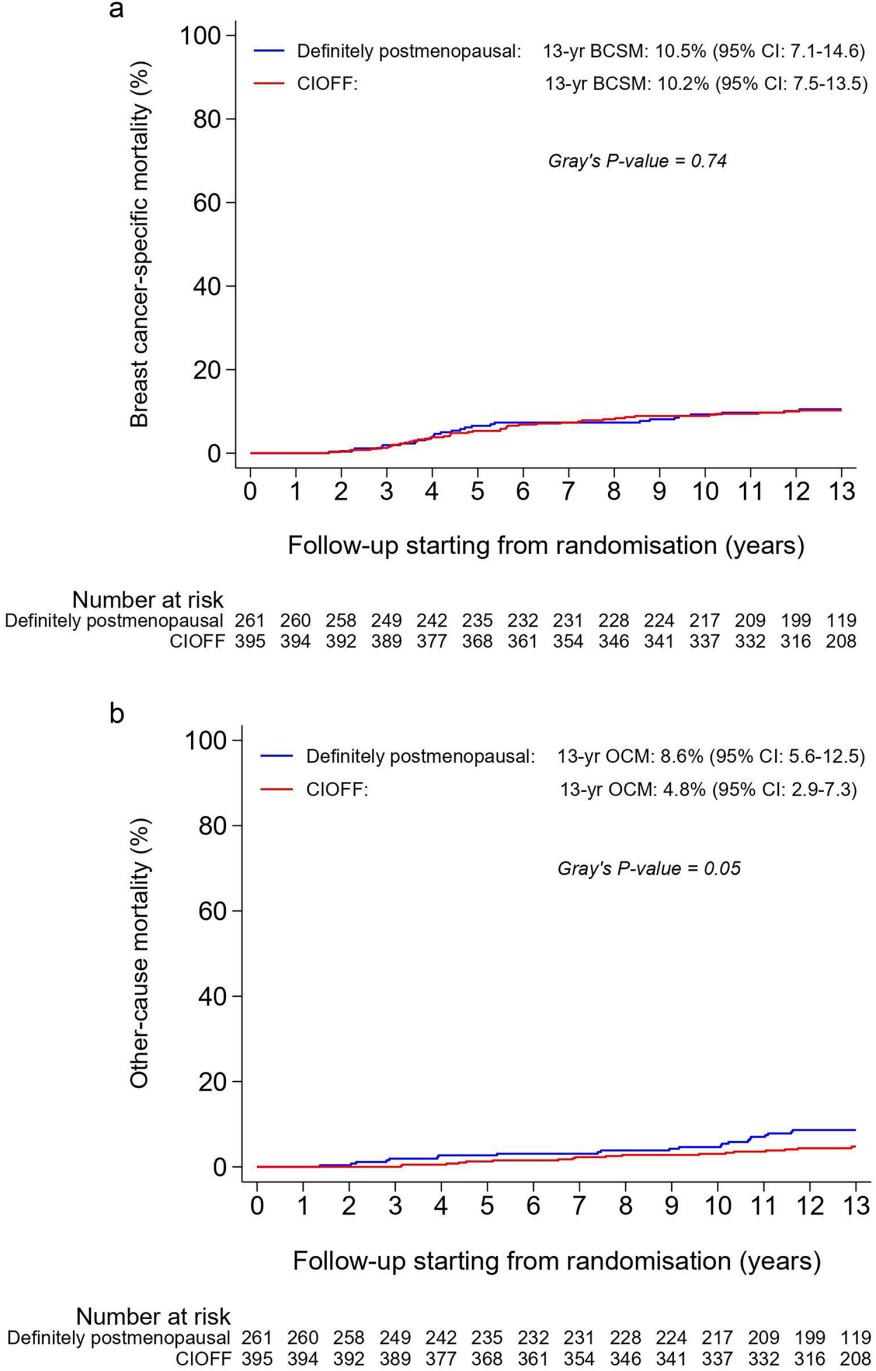
Breast cancer-specific mortality (**a**) and other-cause mortality (**b**) in patients with chemotherapy-induced ovarian function failure (CIOFF) and definitely postmenopausal patients, from randomisation onwards

The 13-year OS rates were, respectively, 85.0% (95% CI 81.0–88.2) and 80.9% (95% CI 75.5–85.2), with an adjusted HR of 0.73 (95% CI 0.50–1.06; *p* = 0.10) (Fig. 2c, Table 2). There was no difference in BCSM (subdistribution (s)HR = 0.93; 95% CI 0.56–1.54; *p* = 0.78) between patient groups (Fig. 3a, Table 2). However, a statistically significant difference in OCM (sHR = 0.48; 95% CI 0.26–0.88; *p* = 0.02) was observed (Fig. 3b; Table 2). In fact, the 13-year OCM rate was 4.8% (95% CI 2.9–7.3) in patients with CIOFF versus 8.6% (95% CI 5.6–12.5) in definitely postmenopausal patients (Fig. 3b).

### Association between OFR and disease outcomes in patients with CIOFF

**Fig. 4:**
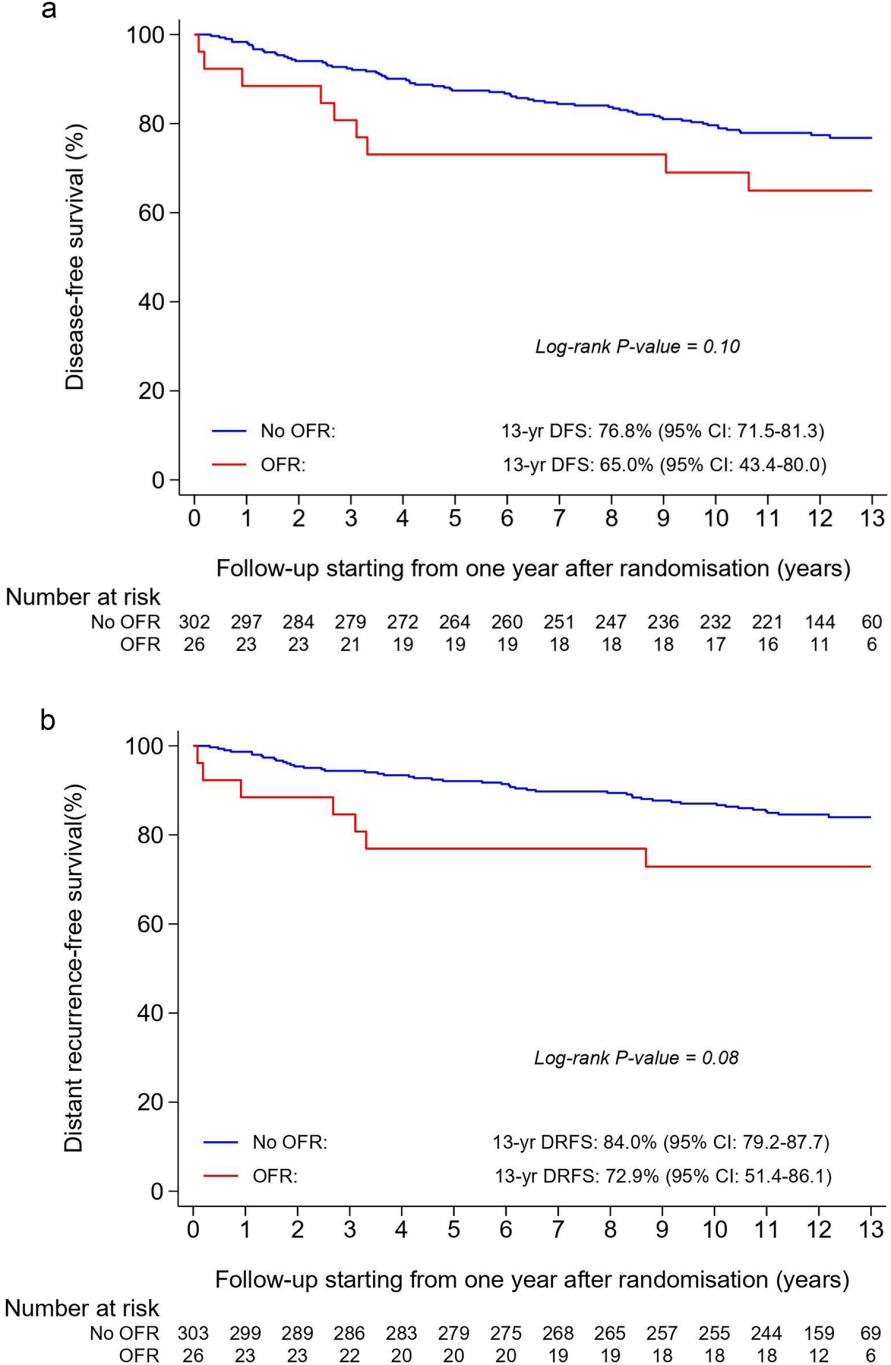

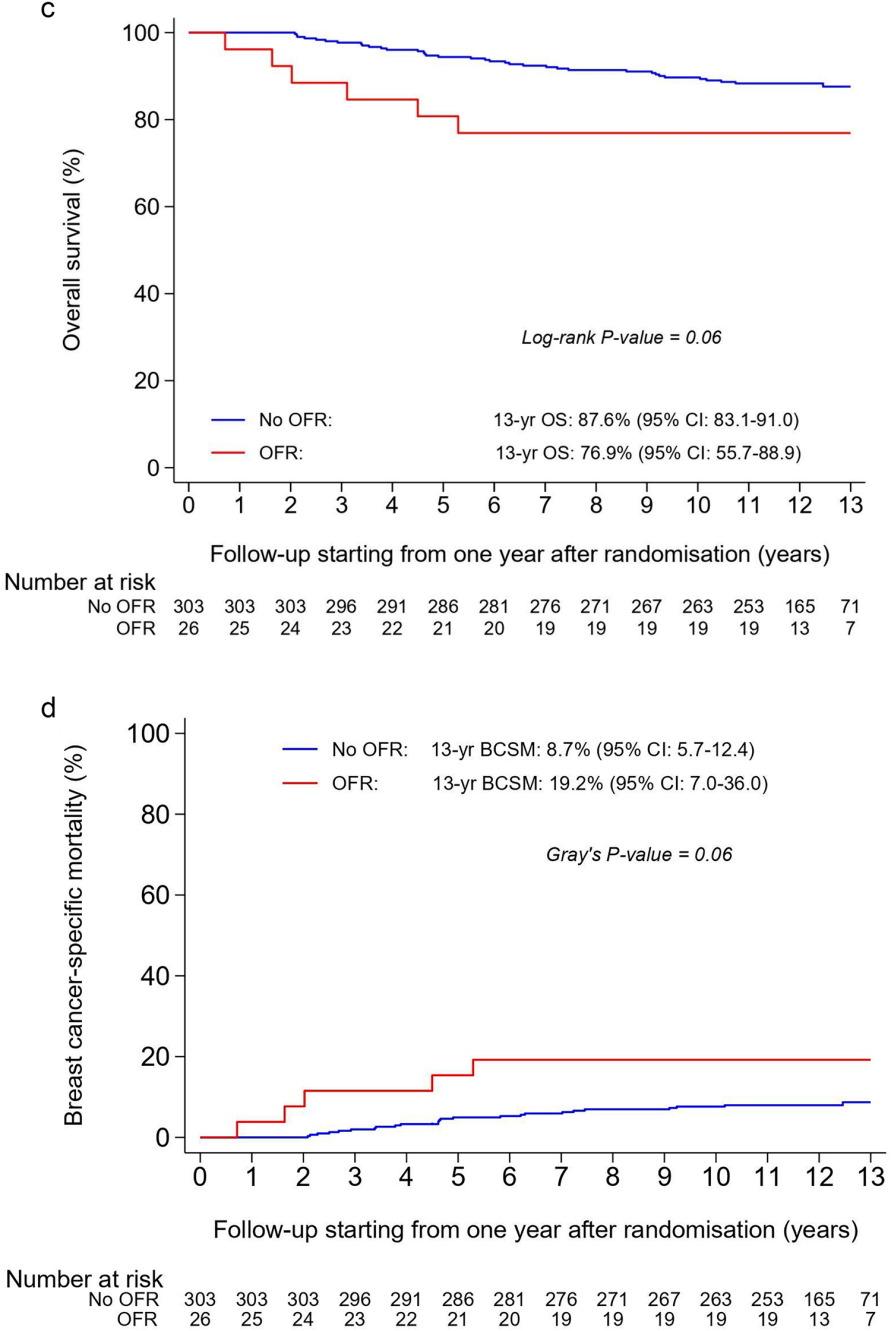
Disease-free survival (**a**), distant recurrence-free survival (**b**), overall survival (**c**), and breast cancer-specific mortality (**d**) in patients with chemotherapy-induced ovarian function failure (CIOFF) who experienced ovarian function recovery (OFR) and patients with CIOFF who did not experience OFR during the first year of treatment with anastrozole, from 1 year after randomisation onwards

Patients with CIOFF who developed OFR experienced a non-statistically significant deterioration in DFS (adjusted HR = 1.54; 95% CI 0.85–2.81; *p* = 0.16), DRFS (adjusted HR = 1.51; 95% CI 0.73–3.11; *p* = 0.26), OS (adjusted HR = 1.64; 95% CI 0.75–3.55; *p* = 0.21), and BCSM (adjusted sHR = 1.98; 95% CI 0.84–4.63; *p* = 0.12) when compared with those who did not develop OFR (Table 2). The survival curves of patients with versus without OFR during the first year of treatment are presented as landmark analyses from one year after randomisation onwards (Fig. 4a–d). The respective 13-year residual survival rates (95% CI) were 65.0% (43.4–80.0) in patients with OFR versus 76.8% (71.5–81.3) in patients without OFR for DFS, 72.9% (51.4–86.1) versus 84.0% (79.2–87.7) for DRFS, and 76.9% (55.7–88.9) versus 87.6% (83.1–91.0) for OS (Fig. 4a–c). The 13-year residual BCSM rate was 19.2% (7.0–36.0) in patients with OFR versus 8.7% (5.7–12.4) in patients without OFR (Fig. 4d).

## Discussion

In this long-term follow-up analysis of 656 patients with HR + BC from the phase 3 DATA study on the extended use of anastrozole, we observed that definitely postmenopausal patients experienced worse outcomes when compared with patients with CIOFF. In-depth analysis showed that this trend towards a worse outcome was not caused by a difference in BCSM, but by a statistically significant difference in OCM. Furthermore, amongst patients with CIOFF, we observed that patients with OFR experienced an increased risk of BCSM when compared with patients without OFR.

Results of our study suggest that definitely postmenopausal patients experience worse DFS, DRFS, and OS when compared with patients with CIOFF. We believe that there are several potential explanations for these differences in outcomes. First, we observed that definitely postmenopausal patients experienced a higher risk of dying from non-BC-related causes. Actually, we revealed that the 13-year OCM rate of patients with CIOFF (4.8%) was half as high as the 13-year OCM rate of definitely postmenopausal patients (8.6%) (sHR = 0.48; 0.26–0.88; *p* = 0.02). These data are important, considering the fact that these women were rather young at randomisation (median age: 51 years). The number of non-BC deaths in our study is too small to perform any additional analyses, but it is known that a younger age at menopause is associated with an increased risk of cardiovascular- and bone-related events [14–20]. Second, differences in BMI should be taken into account. We found that definitely postmenopausal patients were more likely to be overweight or obese at randomisation. Earlier, we reported that overweight and obese patients from the DATA study experienced a significantly worse outcome when compared with normal weight patients, which is in line with the results of several meta-analyses on the adverse prognostic effect of overweight and obesity in patients with BC [21–23]. Maintaining a healthy BMI after becoming postmenopausal is thus important. Third, it is possible that misclassification of menopausal status occurred, as date of last menstruation was self-reported by study participants. We however assume good correlation rates between self-reported and measured dates of last menstruation in the first few years after menopause.

The current analysis shows that OFR during treatment with an aromatase inhibitor is associated with a worse prognosis, even though E2 and FSH levels were measured twice yearly and the majority of patients with OFR received treatment adjustments. It is furthermore important to realise that OFR occurred in a relatively old group of patients who had received chemotherapy more than two to three years earlier. In our study, patients with OFR experienced a clinically relevant decrease in DFS (HR = 1.54; 95% CI 0.85–2.81; *p* = 0.16), DRFS (HR = 1.51; 95% CI 0.73–3.11; *p* = 0.26), and OS (HR = 1.64; 95% CI 0.75–3.55; *p* = 0.21) when compared with patients without OFR during treatment with anastrozole. Patients with OFR also experienced a potentially meaningful increase in BCSM (sHR = 1.98; 95% CI 0.84–4.63; *p* = 0.12). In fact, the 13-year BCSM rate was twice as high in patients with OFR (19.2%) versus patients without OFR (8.7%). Up to this point, only one other prospective cohort study evaluated the association between OFR and disease outcomes in 53 HR + BC patients with CIOFF who received an aromatase inhibitor after initial treatment with tamoxifen [8]. In that study, an adverse association between OFR and DFS (HR = 9.3; 95% CI 3.3–48; *p* = 0.04) was also reported [8]. These findings do not come as a surprise, given the fact that aromatase inhibitors exert negative feedback on the hypothalamus–pituitary–ovary axis, stimulate gonadotropin secretion, and ultimately strongly increase ovarian oestrogen production in premenopausal women. These findings do however once again underscore the importance of OFS in patients with CIOFF who are considered potential candidates for treatment with an aromatase inhibitor. In fact, results of our study suggest that aromatase inhibitors cannot safely be prescribed without adequate OFS, i.e. additional treatment with a GnRH agonist or performance of a bilateral ovariectomy, in patients with CIOFF.

Long-term follow-up results of the SOFT trial showed that premenopausal women with HR + BC who received five years of tamoxifen in combination with 5 years of OFS experienced clinically relevant improvements in DFS (HR = 0.82; 95% CI 0.69–0.98; *p* = 0.03) and OS (HR = 0.78; 95% CI 0.60–1.01; *p* = 0.06) when compared with premenopausal women with HR + BC who received 5 years of tamoxifen monotherapy [24]. The absolute benefit of OFS differed between patient subgroups, showing the greatest benefit in patients who received chemotherapy and patients with “high-risk” clinicopathological features, such as age under 35 years or histological grade 3 tumours [24]. Results of the SOFT trial were corroborated by results of the ASTRRA trial, which showed that premenopausal women with HR + BC who had all been treated with chemotherapy experienced major improvements in DFS (HR = 0.67; 95% CI 0.51–0.87; *p* = 0.003), but not OS (HR = 0.78; 95% CI 0.49–1.25; *p* = 0.31), when receiving five years of tamoxifen in combination with 2 years of OFS versus 5 years of tamoxifen monotherapy [25]. The lack of OS benefit in the ASTRRA trial suggests that 5 years of OFS is superior to 2 years of OFS. A longer duration of OFS may however increase the risk of side effects which may reduce treatment compliance and quality of life. The most optimal duration of OFS is therefore still a matter of debate.

The incorporation of OFS has allowed researchers to explore the potential of aromatase inhibitors in the treatment of premenopausal women with HR + BC [26–28]. It is well known that aromatase inhibitors, either upfront or sequentially after two to three years of tamoxifen, are more effective than tamoxifen in the treatment of postmenopausal women with HR + BC [2]. A recent Early Breast Cancer Trialists’ Collaborative Group (EBCTCG) meta-analysis has now shown that aromatase inhibitors are also more effective than tamoxifen in premenopausal women with oestrogen receptor-positive (ER +) BC who received OFS [29]. In fact, aromatase inhibitors reduced the relative risk of recurrence and distant recurrence by 21% and 17%, respectively [29]. So far, however, no difference in BCSM (rate ratio = 1.01; 95% CI 0.82–1.24; *p* = 0.94) has been observed [29]. The absolute benefit of treatment with an aromatase inhibitor versus tamoxifen in premenopausal women with HR + BC who received OFS is furthermore small, with a 10-year absolute benefit of, respectively, 2.8% for risk of recurrence and 1.9% for risk of distant recurrence [29]. The absolute benefit is expected to be even smaller in patients with a low risk of recurrence. Treatment with an aromatase inhibitor has also been associated with several side effects, such as musculoskeletal symptoms, osteoporosis, or vaginal dryness [28–30]. In patients with low-risk disease, aromatase inhibitors may therefore do more harm than good, especially considering the fact that 12-year OS rates of patients who did not receive chemotherapy exceeded 95% in all three treatment arms of the SOFT trial [24]. These data indicate that appropriate patient selection, considering risks and benefits of OFS, is important.

Our study is the largest study to date assessing the impact of OFR on the disease outcomes of HR + BC patients with CIOFF who received treatment with anastrozole after 2–3 years of tamoxifen. Major strengths of this study include the long-term follow-up period of currently more than 13 years beyond randomisation and the use of data from patients who participated in a randomised controlled trial, in which regular E2 and FSH measurements were performed and patients were consistently monitored during follow-up. This study also has some limitations. Several assays were used to define pre- and postmenopausal E2 and FSH levels across participating hospitals, thereby potentially influencing the incidence of OFR. Guerrero et al. however showed that the incidence of OFR was similar when using two different assays for determining OFR in the same patient [8]. Furthermore, the number of patients with OFR was low, which limited the performance of additional subgroup analyses and impacted the power of our results. A meta-analysis, including all studies on adjuvant endocrine therapy in HR + BC patients with CIOFF, could provide more information on the incidence, prognostic impact, and most optimal treatment of OFR.

In this long-term follow-up analysis, amongst a subset of patients with HR + BC from the phase 3 DATA study, we observed that patients with CIOFF who developed OFR during treatment with anastrozole experienced a potentially clinically relevant deterioration in BC outcomes when compared with patients who did not develop OFR. These findings underscore the importance of adequate OFS in patients with CIOFF who receive treatment with an aromatase inhibitor and support additional research on this topic. A meta-analysis assessing the prognostic impact of OFR in HR + BC patients with CIOFF receiving adjuvant aromatase inhibitor therapy is warranted.

## Supplementary Information

Below is the link to the electronic supplementary material.

Supplementary file1 (PDF 426 KB)

## Data Availability

Data will be shared with interested researchers who are able to provide a methodologically sound proposal with well-defined research questions. Researchers are welcome to contact the corresponding author for more information at vcg.tjan.heijnen@mumc.nl.

## Abbreviations

BC: Breast cancer
BCSM: Breast cancer-specific mortality
BMI: Body mass index
CI: Confidence interval
CIOFF: Chemotherapy-induced ovarian function failure
DFS: Disease-free survival
DRFS: Distant recurrence-free survival
E2: Oestradiol
FSH: Follicle-stimulating hormone
GnRH: Gonadotropin-releasing hormone
HR: Hazard ratio
HR +: Hormone receptor-positive
IQR: Interquartile range
OCM: Other-cause mortality
OFR: Ovarian function recovery
OFS: Ovarian function suppression
OS: Overall survival
sHR: Subdistribution hazard ratio
