# Supplementary material for: Ovarian function recovery in breast cancer patients receiving adjuvant anastrozole treatment: updated results from the phase 3 DATA trial
Source: Breast Cancer Res Treat. 2024 Jun 28;208(1):179–92. doi: 10.1007/s10549-024-07411-w (PMC11452455; doi:10.1007/s10549-024-07411-w)
Supplement: Supplementary file 1 — Supplementary file1 (PDF 426 KB) [file 10549_2024_7411_MOESM1_ESM.pdf]

Supplementary appendix

Supplement to: Lammers, S.W.M., Geurts, S.M.E., Hermans, K.E.P.E. et al. Ovarian function recovery in breast cancer patients receiving adjuvant anastrozole treatment: updated results from the phase 3 DATA trial.

Supplementary Table 1. Endpoint events in the study population (N (%))

	All patients (N = 656)		Patients with CIOFF (N = 329)[a]	
	Patients with CIOFF (N = 395)	Definitely postmenopausal patients (N = 261)	Patients with OFR (N = 39)	Patients without OFR (N = 290)
**Primary endpoint**				
Disease-free survival event[b]	109	84	13	68
Recurrence of the primary tumour	58 (53)	46 (55)	7 (54)	37 (54)
Local recurrence	7 (6)	11 (13)	1 (8)	5 (7)
Regional recurrence	8 (7)	11 (13)	2 (15)	4 (6)
Distant recurrence[c]	49 (45)	32 (38)	7 (54)	30 (44)
Visceral	22 (20)	14 (17)	4 (31)	14 (21)
Bone	34 (31)	18 (21)	5 (38)	19 (28)
Soft tissue	6 (6)	4 (5)	1 (8)	3 (4)
Other	2 (2)	1 (1)	0 (0)	2 (3)
Second primary breast cancer	18 (17)	12 (14)	4 (31)	11 (16)
Second primary cancer	28 (26)	20 (24)	1 (8)	17 (25)
Death without prior breast cancer event	6 (6)	8 (10)	1 (8)	4 (6)
**Secondary endpoints**				
Distant recurrence-free survival event	76	60	9	46
Death from any cause	63	53	8	36
Breast cancer-related death	43 (68)	30 (57)	7 (88)	24 (67)
Non-breast cancer-related death	20 (32)	23 (43)	1 (12)	12 (33)
Second primary malignancy	12 (19)	14 (26)	0 (0)	8 (22)
Cardiovascular disease	1 (2)	4 (8)	0 (0)	1 (3)
Other	7 (11)	5 (9)	1 (12)	3 (8)

Abbreviations: CIOFF = chemotherapy-induced ovarian function failure; E2 = oestradiol; FSH = follicle-stimulating hormone; OFR = ovarian function recovery.

[a]In 66 patients with CIOFF, no follow-up E2 and/or FSH measurements were available to monitor for the incidence of OFR.
[b]Multiple disease-free survival events may have occurred at the same moment.
[c]In some patients, multiple locations of distant recurrence were reported.
